# Complex Connections: Myocardial Fibrosis, Reproductive Factors, and Heart Failure∗

**DOI:** 10.1016/j.jacadv.2023.100698

**Published:** 2023-11-21

**Authors:** Gurusher Panjrath, Andrew D. Choi

**Affiliations:** a Department of Medicine/Cardiology, The George Washington University School of Medicine, Washington, DC, USA; b Department of Radiology, The George Washington University School of Medicine, Washington, DC, USA

**Keywords:** HFpEF, myocardial fibrosis, pregnancy, reproductive factors, women

**Figure:**
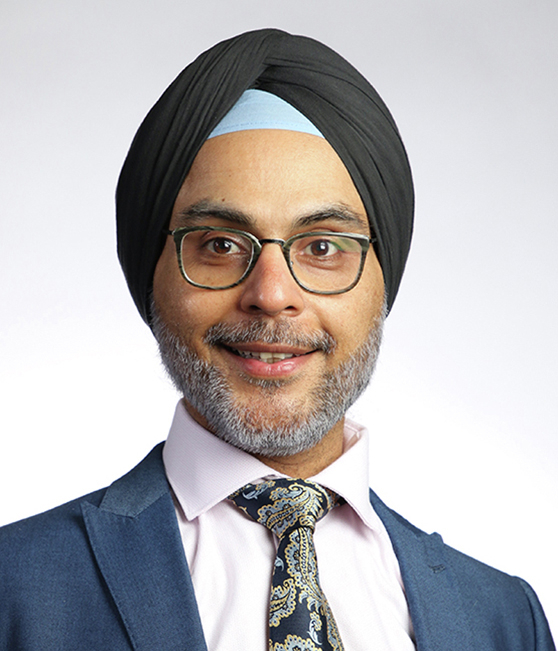

“It’s always the small pieces that make the big picture” —anonymous

Uncoupling the underlying complex and often interrelated pathophysiological mechanisms in the development of heart failure can be challenging. While we understand the higher risk of heart failure with preserved ejection fraction (HFpEF) in women as compared to men, complete understanding of the pathophysiological journey is still missing.^1^^,^^2^ Relationship between reproductive factors and cardiovascular risk over the course of lifetime in women has been complicated by sometimes divergent findings. There exists a need to look at the small and bite size pieces in this puzzle to further unravel the underlying process which might explain the higher prevalence of HFpEF in women. While a lot of attention has been paid to the epidemiological associations between reproductive factors and hormone replacement therapy and risk of developing heart failure, there is a paucity of mechanistic studies. This was likely compounded by lack of robust tools to explore the relationship between individual pieces of this puzzle such as premature menopause, polycystic ovarian syndrome, nulliparity, multiparity, pregnancy loss as well as a shorter total reproductive duration (menarche to menopause), and early or subclinical markers of myocardial remodeling. With progress in cardiac magnetic resonance (CMR) technology and its ability to detect subclinical changes such as myocardial fibrosis and extracellular volumes such explorations have become more feasible. A big picture question to further pose is whether there is a clear link between sub-clinical or clinical heart failure risk and female infertility.

In this issue of *JACC: Advances*, the authors investigated the potential link between reproductive factors with myocardial fibrosis development from the well-established MESA (Multi-ethnic Study of Atherosclerosis) study through CMR using contemporary measures of parametric mapping and myocardial fibrosis.^3^ A total of 596 patients with complete pregnancy history and CMR had further analysis of quantitative native T1 mapping (ms), extracellular volume (ECV [%]) calculation and late gadolinium enhancement imaging on 1.5-T scanners. This was a diverse cohort with 47% non-White individuals including 23% Black. The mean age of the cohort was 67 ± 8 years, 85% had a history of pregnancy and 22% had no live births. A total of 72% reported having gone through menopause, with a mean age of 48 ± 6 years at menopause. This is the first reported study to assess in a diverse cohort comprehensive quantitative and contemporary assessment of the myocardium via CMR and its association with sex specific outcomes.

In comparison to participants with 1 to 2 live births, participants with 0 live births (null gravidity) were found to have higher ECV (%) and native T1 in both unadjusted and fully adjusted models. Those with 0 live births had 0.95% higher ECV and 10.6% higher native T1. There were no differences between those with more than 2 live births and those with 1 to 2 live births. These associations were significant after excluding participants with a history of myocardial infarction, congestive heart failure, and myocardial scar as well as multiple cardiovascular risk factors.

In order to place these findings in context, it is important to first recognize the contemporary role of CMR. Parametric mapping techniques allow for the noninvasive assessment of diffuse myocardial fibrosis that encompass edema, infiltrative disease, iron deposition, and prior infarction.^3^ CMR has had an important role in identifying subclinical and clinical myocarditis in patients with coronavirus-19.^4^ T1 techniques assess the net longitudinal magnetization recovery time of protons after the delivery of a RF pulse. ECV (%) allows for the quantitative assessment of interstitial fibrosis and are affected by vascular volume and blood counts. There is great interest in the field of cardiac imaging and heart failure in the use of T1 and ECV (%) for disease progression tracking in conditions such as cardiac amyloidosis and valvular heart disease.^5^, ^6^, ^7^

How then to explain the findings in the present analysis of MESA? The magnetic effects of iron in hemoglobin may be reduced in anemia that may lead to a linear relationship between blood hemoglobin concentrations and T1 over a range of values. Hypothetically, sex specific factors like menstruation related anemia or even perhaps thrombophilia and hypercoagulability contributing to pregnancy loss could account for prolonged T1 times. Furthermore, the subclinical changes in the myocardium may be associated with underlying auto-immune disorder that may affect the myocardium, but not fully articulated in the cohort. There may be further sex-specific physiologic differences between men’s and women’s hearts that may account for differences in T1 and ECV (%).^8^

While these CMR findings were significant, are they representative of a small piece to the puzzle or a transformational piece for establishing a link between heart failure and reproductive risk? There are several limitations to note. The sample size was small and the MESA questionnaires were self-reported and prone to recall bias. The magnitude of T1 and ECV change was small and while statistically significant may have minor clinical significance. Inherent limitations in the techniques may further limit generalizability. T1 and ECV values have established differences based on the scanner, vendor, site specific normal values and reconstruction techniques (for example MOLLI vs ShMOLLI).^9^ Patient factors such as arrhythmia or respiratory motion artifact may also cause interscan variability. In addition, there may be variability from different myocardial segments (eg, septal vs lateral left ventricle) that should be rectified by careful core laboratory based analysis. ECV (%) calculations should account for appropriate image co-registration from pre contrast and post contrast maps. The finding of prior hormone replacement therapy (HRT) exposure and lower myocardial scar opens up questions on timing of initiation of HRT in this cohort, which is not available in this manuscript. How do we reconcile with other contradicting findings from Womens’ Health Initiative linking HRT use with risk of cardiovascular events or fit in with findings of HRT exposure early post menopause as being cardioprotective? Whether subclinical interstitial myocardial fibrosis or myocardial scar have a differing impact on events need to be further explored.

Perhaps the key question is not to ask about the size of the piece, but how large an opportunity there exists for techniques like CMR to add to our understanding of the field of cardio-obstetrics with many mechanistic questions to be answered beyond epidemiological associations. Our hope is that findings from this study combined with recent evidence on safety of noncontrast CMR during pregnancy at 1.5-T with no exposure to radiation will allow for novel and prospective studies to better understand the effects of pregnancy and other reproductive risks on pathophysiology of HFpEF.^10^^,^^11^

In conclusion, the authors are applauded for first step in defining a small, yet meaningful piece of the puzzle of associations between reproductive factors and subclinical remodeling. We hope that this finding will spur further study in understanding the mechanisms of subclinical cardiomyopathy and clinical heart failure in this under studied group of potentially at-risk women.

## Funding support and author disclosures

The authors have reported that they have no relationships relevant to the contents of this paper to disclose.
